# Supplementary material for: The prevalence and determinants of vitamin D deficiency in Indonesian infants at birth and six months of age
Source: PLoS One. 2020 Oct 5;15(10):e0239603. doi: 10.1371/journal.pone.0239603 (PMC7535980; doi:10.1371/journal.pone.0239603)
Supplement: Attachment — Submitted filename: Response to Reviewers.docx [file pone.0239603.s001.docx]

**The prevalence and determinants of vitamin D deficiency in Indonesian infants at birth and six months of age**

Submission ID: PONE-D-20-14874

Response to reviewers:

|  | Comments | Authors’ response | Change made in the manuscript | Page, line |
| --- | --- | --- | --- | --- |
| **Editor** | |  |  |  |
|  | Please focus introduction on the main subject of study and explain the vitamin D supplementation in infants in details | We have amended the introduction as suggested and added some information related to vitamin D supplementation in the text.  Routine vitamin D supplementation in healthy infants was not standard practice during our study and none of our participants received vitamin D supplementation.  We have added some information on vitamin D-containing food such as infant formula and dietary intake such as egg yolks, red meat and fish | *“Over recent decades, there has been a rapid growth of research interest in perinatal vitamin D deficiency due to a range of potential roles of vitamin D for prevention of disease, such as low birth weight in newborns or infections in early childhood [1-5]. A temperate country such as Australia with a high prevalence of infant vitamin D deficiency, particularly in winter, has implemented preventive measures through routine vitamin D supplementation for high-risk pregnant women and infants (i.e. those with dark skin type and covering clothing), in addition to guidance around safe sun exposure practices [6]. Despite abundant sunlight, vitamin D deficiency is also common in tropical countries, but under-diagnosed. Recent studies have reported that one in every two African and nine in every ten Thai neonates were vitamin D deficient [7, 8]. In tropical regions that experience high neonatal and child health morbidity and mortality, such as in Africa and Southeast Asia [9, 10], sustaining adequate vitamin D level could be a low-cost public health measure to reduce the burden of infectious diseases. However, efforts to prevent, detect, and treat vitamin D deficiency in many of these regions are minimal or absent.*  *Understanding the epidemiology and identification of risk factors for vitamin D deficiency in early life is important for future development of preventive strategies but they are not currently well understood in Indonesia. Indonesia is the most populous country in Southeast Asia and has the fifth-highest global burden of infant morbidity and mortality from acute respiratory infections [11, 12].”*  We have stated this in the discussion section  *“Prenatal and postnatal vitamin D supplementation is not routinely recommended in our setting. Further, there are limited selections of food with a high content of vitamin D in the daily intake of Indonesian people [36]. Only 5% of our study infants had egg yolk in the first six months, mainly those who were living in the rural area, and even less had had fish or red meats. A minority (7%) of mothers reported taking a multivitamin supplement that contained vitamin D during pregnancy with 50% reporting a daily vitamin D intake below 400 IU, the minimum recommended daily dose in pregnancy [6]. None of the participating infants received vitamin D supplementation. Future determination and education on the optimum dose of prenatal and postnatal vitamin D intake or supplementation for the high-risk population in our setting may be warranted.”*  As presented in Table 3 | Page 4-5, line 83 – 135  Page 25-26, line 399-408  Table 3 page 14-15 |
| **Reviewer 1** | |  |  |  |
|  | Supplementation of vitamin D in infants has not been considered thoroughly | During the study, vitamin D supplementation in healthy infants was not routine practice. Vitamin D is expensive and usually only prescribed by a pediatrician under certain medical conditions. None of our participating infants had vitamin D supplementation, but we did collect data on consumption of vitamin D rich foods such as egg yolk, red meat, fish and infant formula. This information is now presented in Table 3 | We have stated this in the discussion section  *“Prenatal and postnatal vitamin D supplementation is not routinely recommended in our setting. Further, there are limited selections of food with a high content of vitamin D in the daily intake of Indonesian people [36]. Only 5% of our study infants had egg yolk in the first six months, mainly those who were living in the rural area, and even less had had fish or red meats. A minority (7%) of mothers reported taking a multivitamin supplement that contained vitamin D during pregnancy with 50% reporting a daily vitamin D intake below 400 IU, the minimum recommended daily dose in pregnancy [6]. None of the participating infants received vitamin D supplementation. Future determination and education on the optimum dose of prenatal and postnatal vitamin D intake or supplementation for the high-risk population in our setting may be warranted.”*  Data on some food that may contain vitamin D are presented in Table 3 | Page 25-26, line 399-408  Table 3 page 14-15 |
|  | The introduction should be summarized | We have amended the introduction | As written in the Introduction section | Page 4-5, line 83 – 135 |
|  | The interpretation of findings should be expanded | We have expanded the findings and study interpretation | We have added discussion on maternal intake of a multivitamin formulation containing vitamin D. | Page 25-26, line 399-408 |
|  | The English writing should be improved | Thank you very much for your feedback. This manuscript has undergone additional review by our English native authors prior to resubmission. The quality of English language is improved. |  |  |
| **Reviewer 2** | |  |  |  |
|  | What data on infant feeding practices was collected, please provide more details, especially regarding the use of infant formula? This is essential information given that infant formula is supplemented with vitamin D. | We collected data on infant formula and some foods such as egg yolk, red meat and fish that contain vitamin D.  This information has now been presented in Table 3 | This additional data is presented in Table 3 | Table 3 page 14-15 |
|  | Please provide the latitude range of the study sites and/or participant locations | We have now added the latitude for the study locations | *“This study was part of the Indonesian Pneumonia and Vitamin D status (IPAD study), a community-based birth cohort study conducted between December 2015 to December 2017 in nine Primary Healthcare Centers and five private clinics in Kota Yogyakarta* ***(7.80° S, 110.4° E)*** *and Kulon Progo regencies* ***(7.83° S, 110.2° E)*** *, Yogyakarta province, Indonesia.”* | Page 5-6, line 148 - 159 |
|  | Given that the focus of the introduction of this manuscript was on infectious diseases, why was there no infectious diseases or any other clinical outcome data included? If this is not to be included, then this manuscript needs an introduction re-write to introduce the content of this manuscript with more relevance. | Thank you for your comment. The infectious disease data (acute respiratory infections) is presented in a separate publication and is not the focus of this manuscript.  We have now rewritten the introduction to focus more on vitamin D prevalence and risk factors |  | Page 4-5, line 83 – 146 |
|  | Lines 54-55 (Abstract): Delete 95% CI 86 - 93% and 95% CI 9-18% as not needed. | We have deleted the 95% CIs. | *“Between December 2015 to December 2017, 350 maternal-newborn participants were recruited and followed up. Vitamin D deficiency was detected in* ***90% (308/344)*** *of cord blood samples and* ***13% (33/255)*** *of venous blood samples at six months.”* | Page 3, Line 54 - 55 |
|  | Lines 124-156: I would recommend that the Vitamin D assay method and definitions of vitamin D deficiency lines 144-147 are combined but move lines 134-137 to be combined with lines 147-156 in a separate section headed Ultraviolet light exposure. | We have combined the vitamin D assay and definition into one section and have now included a separate section that addresses Ultraviolet exposure | ***“Study assays and measurement for vitamin D***  *We measured total serum 25-hydroxyvitamin D3 and 25-hydroxyvitamin D2 concentrations using liquid chromatography tandem mass spectrometry (LC-MS/MS), the current gold standard for vitamin D measurement. The LC-MS/MS analysis was performed by Metabolomics Australia, at the University of Western Australia. The analytical sensitivity (or limit of detection) and the functional sensitivity (or limit of quantitation) of the assay for 25-hydroxyvitamin D3 is 0.5 nM and 2 nmol/L respectively. The center has been certified by the Center of Disease Control and Prevention for standardized vitamin D measurement (r2 =0.99) [18].* ***Vitamin D deficiency was defined as a concentration of 25-hydroxyvitamin D < 50 nmol/L. The vitamin D concentrations at birth and at six months of age were also categorized by severity using the categories suggested by Paxton et al.: mild deficiency (30-49 nmol/L); moderate deficiency (12.5-29 nmol/L) and severe deficiency (< 12.5 nmol/L).[19]”***  **And**  ***“Data acquisition for Ultraviolet B***  *Ultraviolet B (UVB) and Erythema Daily Dose Rate data for the Yogyakarta Special Region were downloaded monthly and obtained from the Aura Data Validation Centre as a part of the National Aeronautics and Space Administration, Goddard Space Flight Centre, Greenbelt, Maryland. (URL link:* [*https://avdc.gsfc.nasa.gov/index.php?site=2057856112&id=79*](https://avdc.gsfc.nasa.gov/index.php?site=2057856112&id=79)*)”* | Page 6-7, Line 174 - 194 |
|  | Line 181: (n= 60)? Should this be n=6 if 344 cord blood samples were collected as stated in line 173? | Thank you for this correction. We have now revised Figure 1 as attached | As displayed in the new Figure 1 | Figure 1 attachment |
|  | Lines 180-185: This Figure 1 description is confusing. A more traditional cohort study participant flow diagram should be included instead. | We have now revised Figure 1 as attached | As displayed in the new Figure 1 | Figure 1 attachment |
|  | Line 190: Delete 95% CI: 86 - 93% as this doesn’t make sense why this needs to be included. | We have deleted the 95% CI | *“The prevalence of vitamin D deficiency was* ***90% in 344 participants*** *at birth and 13% in 255 participants at six months (± 4 weeks) (Fig 2).”* | Page 12, line 265-266 - 272 |
|  | Line 191: Delete 95% CI 9-18% as this doesn’t make sense why this needs to be included | We have deleted the 95% CI | *“The prevalence of vitamin D deficiency was 90% in 344 participants at birth and* ***13% in 255 participants*** *at six months (± 4 weeks) (Fig 2).”* | Page 12, line 265-266 |
|  | Table 1 on participant demographics contains results like exclusive breastfeeding rates, arm and legs uncovered more than half of the outdoor time at six months of age and daytime spent outdoor at six months of age? | Thank you for your feedback.  The information on exclusive breastfeeding rates and sun behavioral has been removed from Table 1 and presented in a separate table (Table 3 – Vitamin D-related factors) | Removed from Table 1  New data listed in Table 3 | Table 1 - Page 10-11  Table 3-Page 14-15 |
|  | Table 1 needs to include season/month of birth | We have added month of birth in Table 1 | Month of birth and season has been listed in Table 1.  We have also added information in the table footnote “**Indonesia is a tropical country with two seasons: dry season (around April to October) and wet season (around November to March**)” | Table 1 - Page 10-11 |
|  | Figure 1: First box should state - 350 participants recruited rather than 350 participants with available blood samples | We have now revised Figure 1 as attached | As displayed in the new Figure 1 | Figure 1 attachment |
