# Supplementary material for: The prevalence and determinants of vitamin D deficiency in Indonesian infants at birth and six months of age
Source: PLoS One. 2020 Oct 5;15(10):e0239603. doi: 10.1371/journal.pone.0239603 (PMC7535980; doi:10.1371/journal.pone.0239603)
Supplement: Attachment — Submitted filename: Response to Reviewers.docx [file pone.0239603.s002.docx]

**The prevalence and determinants of vitamin D deficiency in Indonesian infants at birth and six months of age**

Submission ID: PONE-D-20-14874

**Response to reviewers:**

**Editor**

**Please, rewrite a little introduction as suggested by Reviewer 1.**

**Further suggestions (email correspondence on 3 September 2020)**

Rewrite a little just conclusion of the abstract and to include additional factors which might make this more international like effects of latitude or skin pigmentation (phototype), which could be of interest and the subject of further studies. Please also note that in many countries sun exposure of infants is not advices.

**Author response:**

Thank you very much for your suggestions. We have amended the conclusion in the abstract as suggested and added a few more details in the conclusion in the main body of the paper.

**Change made in the manuscript:**

In equatorial regions, the role of ‘safe’ morning sun exposure in infants and mothers in populations with medium to dark brown skin pigmentation and effective interventions to prevent vitamin D deficiency in newborns and EBF infants, need further consideration and evaluation.(**Abstract’s conclusion section, page 4, line 66 – 69)**

**And**

The role of ‘safe’ morning sun exposure in infants and mothers to prevent early life vitamin D deficiency in populations with medium to dark brown skin pigmentation requires further consideration. **(Main body’s conclusion section, page 28, line 379-381)**

**Reviewer 1**

All comments are successfully considered; however, I suggest that the conclusion section of the abstract would be re-written to be more concise and precise with a message for international readers.

**Author response:**

Thank you for your suggestion. We have amended the abstract’s conclusion as suggested and added a few more details in the main body’s conclusion.

**Change made in the manuscript:**

In equatorial regions, the role of ‘safe’ morning sun exposure in infants and mothers in populations with medium to dark brown skin pigmentation and effective interventions to prevent vitamin D deficiency in newborns and EBF infants, need further consideration and evaluation.(**Abstract’s conclusion section, page 4, line 66 – 69)**

**And**

The role of ‘safe’ morning sun exposure in infants and mothers to prevent early life vitamin D deficiency in populations with medium to dark brown skin pigmentation requires further consideration. **(Main body’s conclusion section, page 28, line 379-381)**
